# Round the Hospitals

**Published:** 1920-08-07

**Authors:** 


### ROUND THE HOSPITALS.

Princess Beatrice opened a three-days' bazaar and fête on July 22 at Northwood House, Cowes, in aid of the Frank James Memorial Cottage Hospital, Cowes, of which she is president. Extension is urgently required, and a ward with five new beds is in course of construction. The hospital is highly popular in the neighbourhood, and the skill and friendliness of the matron and nursing staff are much appreciated by the patients. A wheeled ambulance has been presented by the B.R.C.S. and the local voluntary-aid detachment. A week later Princess Beatrice graciously drove across the island to open formally the House of Rest at Bonchurch for trained nurses, provided by Mr. and

**ROUND THE HOSPITALS**—(*continued*).

Mrs. Martin Harvey and the British Women's Hospital Committee. The house is situated in romantic surroundings within a couple of miles of Ventnor. Great wooded cliffs tower up behind, leaving a narrow front where a cluster of houses and cottages look out over the sea, undisturbed by traffic of any description, for the main road runs high above. It is doubtful if a more charming and reposeful situation exists in the British Isles, and the soft climate makes it particularly suitable for restoring strained nerves and over-taxed energies. There are eight beds, and any fully-trained nurse who requires to recuperate after illness is eligible at a payment of 21s. per week, inclusive. All applications should be made to Miss Hall, Secretary of the Nation's Fund for Nurses, 32 North Audley Street, London, W. 1, but there are no vacancies till the end of October.

---

The long delay which has in some cases taken place in demobilising Army nurses who will obviously never be fit for duty again is to be ended. The Army Council announces that members of Q.A.I.M.N.S. Reserve and T.F.N.S., as well as V.A.D. nursing members and special military probationers, who are permanently unfit for further service, will be forthwith demobilised. Nurses who are classed as temporarily unfit owing to their military service will be demobilised either at the end of twelve months' absence from duty on account of medical unfitness, or on ceasing to require indoor hospital treatment, whichever happens first. Nurses belonging to the Services who are temporarily unfit by reason of disability not caused by military service will be demobilised after three months' absence from duty, or as soon as they cease to require indoor hospital treatment. This is another step towards restoring the Services to pre-war conditions. It serves no good purpose to keep on the establishment nurses who are unable to resume their duties, but may when set free find openings for usefulness in other directions.

---

From September 1 members of the R.A.F. Nursing Service are to wear new uniform and hat badges. The former badge is of two patterns, one a winged Caduceus of Mercury surmounted by a crown, made in "all gilt," for outdoor uniform, and the other of a similar pattern, but with silver wings, for mess uniform. The hat badge is in future to be the same as that worn by officers of the R.A.F., with a plain black mohair band.

---

Aberdeen and Glasgow, Guy's and St. Bartholomew's Hospitals have the honour of having trained the four successful candidates for the Cowdray and College studentships at King's College for Women, Campden Hill. Miss Isabella Stewart, from the Royal Infirmary, Aberdeen, and Miss E. M. Yates, from Guy's Hospital, gain the Cowdray studentships for sister-tutors, while Miss F. E. Dunn, from St. Bartholomew's Hospital, and Miss M. D. Smith, from the Victoria Infirmary, Glasgow, gain the College studentships for health visitors. The conditions attaching to these studentships and the ground covered by the courses, which extend to one year, from October next, were given in full in our issue of May 15 last, pages 174-5.

---

It is hardly credible that Birmingham can allow the District Nursing Association, which has for fifty years ministered to its sick poor, to reduce its staff by half for lack of means. Nevertheless, at the last annual meeting it was reported that a crisis had been reached in its affairs; only 360 subscribers support its operations, and their contributions, totalling only £750, are quite inadequate. Only a few out of the thirty districts can now be covered, and two out of the three homes are threatened. A grave warning has been given by Miss Musson of the Birmingham General Hospital, writing in the *Birmingham Post*. Already, she points out, the aged and chronic invalids are perforce neglected; patients discharged from hospital before they are really convalesecnt in order to make room for other urgent cases must depend on the district nurses for the completion of their recovery, and the whole chain of relief to the sick will be endangered if the Nursing Association can no longer supply this care. Miss Musson claims the gratitude of the city for the devotion of the superintendents and nurses of Q.V.J.I.N., "often greatly overworked, always most inadequately paid," and writes warmly of the senior superintendent, who has given devoted service to the city for the last quarter of a century, almost unknown except in the poorer quarters. We cannot doubt that the public-spirited in Birmingham will rally at the call and come to the rescue of the Association.

---

A novel birthday party was held at the Royal Hampshire County Hospital on July 19 to celebrate a year's work in the new maternity ward. All the babies born in the ward during the year, to the number of 126, were invited, and no fewer than eighty were present with their mothers. A great many distinguished people, including Lady Portal, after whom the ward is named, gathered to meet them. The hostess was the matron, Miss Carpenter Turner, assisted by Sister Ball of the maternity ward and other members of the staff, and she gave a charming little address of welcome to the guests. Dr. Wilkes, Deputy Medical Officer of Health, and Dr. Smythe made excellent speeches and explained the aims and value of the newly instituted maternity department. They are able to train four nurses in midwifery every three months, and supplement their cases by co-operation with the Winchester Maternity Society. An ante-natal clinic is run in connection with the maternity department.

---

General appeals are seldom very successful, because everyone is inclined to think they are meant for any but themselves. We regret particularly that so few have taken the words of Lady Astor about

**ROUND THE HOSPITALS—**(*continued*).

outings for the disabled to heart. The response was so small, she has again written to rouse the public to a sense of their obligations towards the soldiers still lingering on in hospital and feeling very keenly the monotony and sense of hope deferred, and Major-General Sir F. Maurice strongly endorses her appeal. The right person to approach with offers of drives or money to provide drives is the Director, Major-General Sir Richard Ewart, at the London County Council offices. Many are very bad cases, men without legs or arms, or spinal cases hardly able to move. Perhaps some of our readers can assist by turning the general appeal to an individual one, and bringing the great need of our wounded men to the notice of such as can lend their cars.

---

Miss Ripley, matron of the Gloucester Royal Infirmary and Eye Institution, was well inspired in the garden fête and pound day held on her initiative a short time ago, the first festivity of the kind in the annals of the charity. She and her nurses worked hard to enlist support, and themselves, with the patients' help, furnished an attractive stall. The Mayor and Mayoress, with many friends of the hospital, rallied to the good cause, and the result was a sale which, with sideshows, brought in over £200. In expressing her thanks in a letter to the Press, the matron declared that the main object of the fête had been to rouse a real personal interest in the infirmary. In this we believe she sounded the right note. Experience shows that such reunions, when the wards and other departments of the hospital are open to inspection, do more to promote a sense of responsibility towards the sick than thousands of urgent appeals. They help to build up that strong circle of personally interested friends without which voluntary hospitals are bound to languish.

---

The last of the little band of pioneer nurses, twelve in number, who had the distinction of being Florence Nightingale's first group of probationers, has passed away at Nottingham at the age of eighty-five. Mrs. Batten (Sister Eliza Trueman) was trained while still young, and devoted herself to workhouse nursing at Liverpool under Miss Agnes Jones, who, it will be remembered, gave all her wealth and energies to introduce trained nursing into these institutions. Miss Jones died in 1868 from fever contracted in the course of her labours, and Miss Nightingale wrote after the news had reached her a very touching letter to all the nurses who worked under her at Liverpool, of which Sister Trueman always preserved her copy. Miss Trueman continued nursing till she reached the age of sixty, when she returned to Nottingham, her native city, and four years later married Mr. Abraham Batten.

---

Nurses, health visitors, and social workers should note the series of eighteen lectures on tuberculosis which will be given at the Brompton Hospital for Consumption and Diseases of the Chest, on Tuesdays and Fridays, from October 12 to December 10, at 8 p.m. The fee for the course is £1 1s., for single lectures 2s., and for the course with demonstrations and examinations, £2 2s. A complete list of the lectures is published on p. 494. They are specially designed to be of great help in the nursing and management of tuberculosis both in hospital and home, and include lectures on the history of tuberculosis, on its economic factors, and on the principles of social work.

---

An effort is being made in Hertfordshire to organise a reserve staff composed of V.A.D. nurses for use in epidemics. The County Council has appointed special nurses whose services will be available for the nursing of measles, infantile diarrhœa, puerperal fever, or other acute illness. The county M.O.H. reports that Herts is now supplied with a complete service of midwives. The standard of midwives' work is generally high, but the attendance at a series of lectures to midwives was disappointing, though the work of the midwives is so important in relation to the welfare of the mother and the prevention of infant mortality, that no occasion should be missed by them to increase their knowledge and raise their standard of efficiency. The infant mortality in the County of Herts last year was 49.5 per 1,000, which is the lowest yet reached. The Health Committee has under consideration a proposal to make a yearly grant of £10 to the district nurses and health visitors to cover the cost of uniform.

---

A charming fête was given recently at Peak Hill Lodge, Sydenham, when the new home of rest for mothers with babies was opened by Lady Forster accompanied by her husband, Lord Forster, the recently appointed Governor-General of Australia. The home is intended to form a second line of defence for mothers in south-east London, to recuperate after a bad confinement or build-up their strength after hospital treatment. The house has been given for the purpose by Lord Forster, it is well placed in its own grounds and has been entirely re-drained. It has been open for some time and hitherto Lady Forster has found the funds for its maintenance, but people of Sydenham are now invited to help. There can be little doubt that this excellent undertaking will be carried on during the absence of the founders at the Antipodes in the spirit in which it was started.

---

The Kent Education Committee in consultation with the County Medical Officer of Health offers twenty-four midwifery scholarships tenable only at approved institutions. Fourteen candidates are to be nominated by the Kent County Nursing Association for women who will practise in the county, and six by the County Medical Officer, the remaining four by either one or other of these authorities. These scholarships will cover the fee payable to the institution selected for training, but will not in any case exceed £35. In addition, six post-graduate scholarships, of a value not exceeding £16 are offered to trained midwives already in practice to enable them to bring their knowledge up-to-date.

